# Association of Histological Features of Cortical and Medullary Peritubular Capillaries With Progressive CKD

**DOI:** 10.1053/j.ajkd.2026.05.010

**Published:** 2026-07-18

**Authors:** Jaidip M. Jagtap, Aleksandar Denic, Yijiang Chen, Nikolas E.J. Schmitz, Afsana A. Shaik, Fnu Aperna, Mahesh Kumar, Syed Khooshal Fareeduddin, Aidan F. Mullan, Bradley J. Erickson, Vidit Sharma, Martin T. Strauch, Andrew R. Janowczyk, Peter Boor, Laura Barisoni, Andrew D. Rule

**Affiliations:** Division of Nephrology and Hypertension, Mayo Clinic, Rochester, Minnesota; Department of Radiology, Mayo Clinic, Rochester, Minnesota; Division of Nephrology and Hypertension, Mayo Clinic, Rochester, Minnesota; Department of Radiation Oncology, Stanford University, Stanford, California; Institute of Pathology, University Clinic Aachen, RWTH Aachen University, Aachen, Germany; Division of Nephrology and Hypertension, Mayo Clinic, Rochester, Minnesota; Division of Nephrology and Hypertension, Mayo Clinic, Rochester, Minnesota; Division of Nephrology and Hypertension, Mayo Clinic, Rochester, Minnesota; Division of Nephrology and Hypertension, Mayo Clinic, Rochester, Minnesota; Division of Clinical Trials and Biostatistics, Mayo Clinic, Rochester, Minnesota; Department of Radiology, Mayo Clinic, Rochester, Minnesota; Department of Urology, Mayo Clinic, Rochester, Minnesota; Institute of Pathology, University Clinic Aachen, RWTH Aachen University, Aachen, Germany; Department of Biomedical Engineering, Georgia Institute of Technology and Emory University, Atlanta, Georgia; Department of Oncology, Division of Precision Oncology, Geneva University Hospitals, Geneva, Switzerland; Department of Diagnostics, Division of Clinical Pathology, Geneva University Hospitals, Geneva, Switzerland; Institute of Pathology, University Clinic Aachen, RWTH Aachen University, Aachen, Germany; Department of Pathology, Division of AI and Computational Pathology, Duke University, Durham, North Carolina; Department of Medicine, Division of Nephrology, Duke University, Durham, North Carolina; Division of Nephrology and Hypertension, Mayo Clinic, Rochester, Minnesota

## Abstract

**Rationale & Objective:**

Peritubular capillary (PTC) rarefaction occurs in chronic kidney disease (CKD), but the independent association of PTC histological features with CKD progression is uncertain. This study assessed this relationship.

**Study Design:**

Historical cohort study.

**Setting & Population:**

Patients who underwent radical nephrectomy for a kidney tumor.

**Exposure:**

PTC histological features including density, mean area, major axis length, and circularity were defined in the cortex (excluding regions of interstitial fibrosis and tubular atrophy [IFTA]) and medulla using previously developed deep learning models.

**Outcome:**

Progressive CKD was defined as the composite of dialysis, kidney transplantation, a sustained decline in estimated glomerular filtration rate (eGFR) of ≥30%, or a sustained eGFR <10 mL/min/1.73 m^2^ that was ≥5 mL/min/1.73 m^2^ lower than the postnephrectomy value.

**Analytical Approach:**

Cox proportional hazards analysis where follow-up was censored at death, cancer recurrence, or the last serum creatinine value adjusted for chronic histological changes (IFTA, glomerulosclerosis, arteriosclerosis, and arteriolar hyalinosis) and the CKD risk factors of age, sex, body mass index, hypertension, diabetes, eGFR, and proteinuria.

**Results:**

Progressive CKD was experienced by 94 patients (6.1%) of 1,553 followed for a median of 4.3 years. A mean of 28,055 PTCs and 332 glomeruli were detected in each wedge section. In cortical and medullary tissue, a lower density of PTCs and PTCs that were more circular and less elongated were associated with progressive CKD in analyses adjusted for kidney function and CKD risk factors. After additional adjustment for chronic histological changes, only cortical PTCs that were more circular and less elongated were associated with progressive CKD.

**Limitations:**

Study limited to patients who underwent a radical nephrectomy for a tumor.

**Conclusions:**

Reduction and simplification of the microvasculature in nonscarred regions of cortex and medulla are associated with progressive CKD.

Evaluation of the changes in chronic kidney disease (CKD) has historically focused on interstitial fibrosis, tubular atrophy, glomerulosclerosis, and arteriosclerosis, but recent studies have highlighted the role of the microvasculature for disease progression.^1–6^ Peritubular capillaries (PTCs) are crucial for oxygen transport, nutrient exchange, and waste elimination in the kidney.^7,8^ However, precise assessment of the status of microvasculature cannot be achieved by the human eye.^1,2,9,10^ Rarefaction of PTCs, rounder shape, and shorter major axis as well as more complex boundary patterns (eg, Fourier descriptors) have been described to be associated with CKD.^1–6^ Many prior studies of PTCs have relied on immunohistochemistry analysis, have been limited to small cohorts with small tissue specimens, have included only specific kidney diseases, have lacked longitudinal follow-up for clinical CKD outcomes, or had limited adjustments for other clinical and pathological characteristics known to be associated with CKD.

The added prognostic value of PTC features (including density) beyond established histologic changes of chronicity such as interstitial fibrosis and tubular atrophy (IFTA) is unclear. In particular, arteriosclerosis with intimal thickening of arteries and hyalinosis of arterioles and the loss of capillary loops with glomerulosclerosis may lead to progressive CKD along the same pathway as PTC rarefaction. In clinical practice, specimens sometimes only contain medulla. Established histologic changes of chronicity used to assess CKD severity are typically measured in the cortex, and visual assessment of the medulla has limited value.^11,12^ Whether PTC features in the medulla (vasa recta) can be used to assess chronicity of CKD warrants study.

This study applied a previously developed deep learning (DL) model to images of large wedge sections of kidney tissue to segment the PTC profiles^13^ within the interstitium^14^ of cortex (excluding IFTA) and medulla. The objectives of this study were to determine whether PTC features are associated with progressive CKD in each of these kidney regions and whether the associations detected were independent of other chronic histological changes or of kidney function and CKD risk factors.

## Methods

### Study Population

We studied patients from a previously described cohort within the Aging Kidney Anatomy (AKA) study^15^ who underwent a radical nephrectomy for a renal tumor at Mayo Clinic, Minnesota, between 2000 and 2021, and were followed until April 2024. The following were used as exclusion criteria: no serum creatinine testing after the nephrectomy and during the subsequent 4 months; cancer recurrence, kidney failure, or death within 4 months after surgery; a specific kidney disease on histology (other than mild–moderate diabetic nephropathy); severe/diffuse interstitial inflammation; severe ischemia with percentage of interstitial fibrosis and tubular atrophy (IFTA) > 90% of cortex; a large focal scar; or end-stage kidney histology (thinned and nearly completely scarred cortex).^15^ This study was approved by the Mayo Clinic Institutional Review Board (14–005798) with a waiver of consent because data were limited to medical records and clinically obtained tissue specimens.

### Clinical Assessment

Baseline clinical characteristics were obtained from medical records before the nephrectomy, as previously described elsewhere.^15^ This included age, sex, race, body mass index, diabetes mellitus, hypertension, creatinine-based estimated glomerular filtration rate (eGFR) using the 2021 CKD Epidemiology Collaboration equation^16^ and estimated 24-hour urine protein from spot urine protein to osmolality ratios.^17^ Hypertension was defined by clinical diagnosis of hypertension on review of medical records at the time of nephrectomy. The 24-hour urine protein was determined by estimation from spot protein-to-osmolality ratios.^17^

For longitudinal analyses, the baseline was defined as occurring at 4 months after nephrectomy, and the baseline eGFR was determined using the last available serum creatinine before this 4-month time point. This allowed the study of eGFR loss relative to the new baseline after the nephrectomy rather than eGFR loss due to the nephrectomy itself.

Per a standardized clinical protocol for long-term monitoring after the nephrectomy, patients returned for follow-up visits every 3 to 6 months during the first year and then every 6 to 12 months subsequently. This visit included a measurement of serum creatinine levels. For patients who did not return for a follow-up visit, attempts were made to contact them by phone to determine their vital status. If they were alive, they were surveyed or had outside medical records reviewed for recent serum creatinine levels, dialysis, and kidney transplantation.

Progressive CKD was defined as the composite of dialysis, kidney transplantation, a 30% or more decline in eGFR sustained for at least 3 months, or an eGFR < 10 mL/min/1.73 m^2^ that was at least 5 mL/min/1.73 m^2^ below baseline and sustained for at least 3 months. The follow-up period ended in April 2024.

### Kidney Histology and Manual Annotations

A large wedge section (far from the tumor) of the formalin-fixed whole kidney specimen was embedded in paraffin. A 2- to 3-μm-thick section was cut from the paraffin-embedded tissue block, periodic acid-Schiff (PAS) stained, and scanned into a high-resolution (×400) digital whole-slide image (WSI) (Aperio scanners; Leica Microsystems). The WSIs were annotated by investigators at the Mayo Clinic who were masked to clinical characteristics. Training data and reproducibility studies were used to ensure consistency between different investigators. Quality control of the annotations was performed to ensure accuracy and consistency.

The WSIs were presented with ImageScope software (version 12.2.2.5015; Aperio; Leica Microsystems) on a large touchscreen tablet. The cortex, medulla, and IFTA foci profiles (clusters of at least 3 atrophic tubules with surrounding fibrosis) in the cortex were manually traced (Fig 1A). The transition boundary from cortex to medulla was determined at low magnification using the location of arcuate arteries and the presence versus absence of glomeruli. The %IFTA (area of IFTA divided by area of cortex) and IFTA foci density (number of IFTA foci divided by area of cortex) were calculated.^18,19^ All nonsclerotic glomeruli (NSG), all globally sclerotic glomeruli (GSG), and the intima and lumen of the 3 most orthogonal arteries to the plane of the wedge section were manually traced. The total number of glomeruli and globally sclerotic glomeruli (GSG) was used to calculate the %GSG. Weibel-Gomez stereological models were used to calculate the glomerular volume for each patient.^20^

Arteriosclerosis was assessed by artery % luminal stenosis from intimal thickening as calculated from the area of intima divided by the areas of intima and lumen using the mean of the 3 traced arteries. Arteriolar hyalinosis was identified in arteriole profiles (at least 500 μm apart to avoid counting the same arteriole more than once) and reported as a density (number per area of cortex).

### DL Model and Image Processing

We used a previously validated DL model that had been developed using the Nephrotic Syndrome Study Network (NEPTUNE) to segment PTCs.^13^ The PTC model was previously validated against CD34 staining.^2^ In addition, we used another DL model (FLASH) to segment the interstitium.^14^ The FLASH model was first optimized by fine-tuning using 20 WSIs from our dataset. The PTC model was applied to the WSI, and analyses were performed separately in the interstitium of the cortex (excluding regions of IFTA) and in the interstitium of medulla (Fig 1).

PTCs that were not within the interstitium were considered false positives and excluded from analysis. Further, thresholds of <10 μm^2^ or >350 μm^2^ were used to exclude false positive PTC profiles (manual review using Patch Sorter revealed these were usually not PTCs).^21^

To assess the validity of the DL models for detecting PTCs, we analyzed 10 random patients in our cohort. At low magnification, we placed 100,000 μm^2^ circular regions of interest in a non-IFTA cortex region for each patient. The PTCs in each region were counted by the DL model and by a visual inspection (A.D.) masked to the DL model results. The bias of the DL model compared with visual inspection within the same region was determined with paired *t*-tests.

### PTC Features

The PTC features assessed were the mean area, count density, area density, circularity, eccentricity, major axis, and aspect ratio (major axis divided by minor axis). Examples of the variability in PTC features are shown in Figure 2. As tubular profile size associates with progressive CKD,^15^ the PTC count density and area density were indexed to the interstitium area for each region: non-IFTA cortex and medulla. The formulas used for PTC feature calculations are provided in Item S1.

### Statistical Analysis

The risk of progressive CKD with PTC features was assessed using Cox proportional hazards models with censoring at death, cancer recurrence, or last serum creatinine value. Models were evaluated unadjusted, adjusted for chronic histological changes in the cortex (%GSG, %IFTA, IFTA foci density, artery % luminal stenosis, and arteriolar hyalinosis density), and adjusted for baseline CKD risk factors and kidney function (age, sex, body mass index, hypertension, diabetes, eGFR, and proteinuria). Analyses adjusting for chronic histological changes were done to evaluate whether the PTC features provided prognostic information independent of established chronic changes on histology known to predict progressive CKD. Analyses adjusting for CKD risk factors and kidney function were done to evaluate whether PTC features provided prognostic information beyond the clinical evaluation (not including kidney histology) that is often used to predict progressive CKD.

Additional models using backward stepwise selection (starting with all PTC features but excluding the least significant PTC feature one at a time until only statistically significant features at *P* < 0.05 remained) identified the PTC features that predicted progressive CKD within the same model. Cubic splines assessed for nonlinearity in the prediction of progressive CKD by PTC features. The PTC features were further assessed for correlation with each other and with baseline clinical characteristics with Spearman’s correlation coefficient. Using linear regression models, we assessed whether PTC features in the medulla could predict %IFTA in the cortex.

## Results

### Cohort Characteristics

There were 1,553 radical nephrectomy patients (mean age, 63.8 years; 64.6% men) with a mean postnephrectomy baseline eGFR of 51 mL/min/1.73 m^2^ and 24-hour urine protein of 161 mg studied (Table 1). The median follow-up time was 4.3 (IQR, 2.1–8.2) years, and 94 patients (6.1%) developed progressive CKD (1 underwent kidney transplantation, 10 started dialysis, 15 had sustained eGFR < 10 mL/min/1.73 m^2^, and 68 had a sustained eGFR decline > 30%.

### PTC Model Validation

There was no meaningful difference in the PTC counts in the regions of interest between the DL model and visual counting for non-IFTA regions of cortex (bias, 0.4 PTCs [95% CI, −2.5 to 3.3]).

### PTC Features Across Non-IFTA Cortex and Medulla Regions

There were a mean 28,055 PTCs per WSI. The PTCs in the medulla (vasa recta) were larger (63.5 vs 54.1 μm^2^, *P* < 0.001) but with lower count density (0.00030 vs 0.00041 per μm^2^, *P* < 0.001) compared with the PTCs in the cortex (Table 2). The PTC count density was assessed for correlation with the interstitium area in the cortex and medulla (*r*_s_ = 0.01, *P* = 0.7 and *r*_s_ = −0.27, *P* < 0.001, respectively). The PTC count density was correlated between the cortex and medulla (*r*_s_ = 0.51, *P* < 0.001).

### PTC Features and Risk of Progressive CKD

In the cortex, smaller PTC area was associated with progressive CKD but not after adjusted analyses (Table 3). Also, lower PTC count density and area density were associated with progressive CKD. The association with lower PTC count density remained significant after adjustment for kidney function and CKD risk factors but not after adjustment for chronic histological changes. Higher PTC circularity and shorter major axis were associated with progressive CKD in unadjusted and adjusted analyses. In the medulla, lower PTC count density and area density were associated with progressive CKD, and the association with lower PTC area density remained after adjustment for kidney function and CKD risk factors. When measured in the medulla, shorter PTC major axis was associated with progressive CKD after adjustment for chronic histological changes in the cortex. Figure 3 shows the relatively linear relationships between PTC features and the risk of progressive CKD.

Table S1 shows the correlations between different PTC features in the cortex and medulla. In the cortex, PTC count density and PTC area density were highly correlated (*r*_s_ = 0.96, *P* < 0.001). The PTC major axis length negatively correlated with PTC circularity (*r*_s_ = −0.47, *P* < 0.001) and positively correlated with PTC count density (*r*_s_ = 0.37, *P* < 0.001) (Fig S1). In the same model, lower PTC count density (HR, 0.72 per SD, *P* = 0.003) and more circular PTCs (HR, 1.32 per SD, *P* = 0.006) independently predicted progressive CKD. Figure 4 shows examples of 2 patients with <5% IFTA in the cortex but with different risks for progressive CKD based on their PTC features.

### Clinical and Histological Correlations With PTC Features in the Non-IFTA Cortex

Table 4 shows that high-risk PTC features (lower PTC count density, shorter PTC major axis, and higher PTC circularity in the non-IFTA cortex) correlated with older age, reduced kidney function (lower GFR and higher proteinuria), and more IFTA. Lower PTC count density and shorter PTC major axis also correlated with higher body mass index, hypertension, higher %GSG, and higher artery % luminal stenosis. Lower PTC count density further correlated with male sex, larger glomerular volume, and more arteriolar hyalinosis. Men also had longer PTC major axis and less PTC circularity than women.

### Estimating %IFTA in the Cortex From PTC Features in the Medulla

The %IFTA in the cortex showed negative correlation with the PTC count density in the medulla (*r*_s_ = −0.19; *P* < 0.001) and PTC circularity in the medulla (*r*_s_ = −0.10; *P* < 0.001). The cortical %IFTA could only be weakly estimated from PTC count density in the medulla (R^2^ = 0.039) (Fig S2).

$$\begin{array}{r} \mathrm{Predicted}\;\text{\%}\;\text{IFTA}\;\text{in}\;\text{cortex}\;=\text{exp}(0.60-1,928\\ \left.\times \left[\text{Medulla}\;\text{PTC}\;\text{count}\;\text{density}\;\left(\mu {\text{m}}^{-2}\right)\right]\right) \end{array}$$

Adding PTC circularity to this regression model only modestly improved the model fit (R^2^ = 0.041). Likewise, adding sex to this regression model only modestly improved the model fit (R^2^ = 0.047).

## Discussion

Our study contributes to the current literature by detailing PTC features and their clinical relevance in a large cohort of patients who underwent a radical nephrectomy. Leveraging digital and computational pathology for the automated segmentation and characterization of PTC morphology, we demonstrated in the non-IFTA cortex that PTC rarefaction (decreased density) along with increased PTC circularity and shorter major axis length was predictive of progressive CKD and associated with more severe IFTA, glomerulosclerosis, proteinuria, and lower eGFR.

Prior studies have shown that PTC features related to shape and spatial distribution are associated with CKD in various patient populations.^1–3,6^ Specifically, investigators have reported PTC loss and shape changes—from elongated to rounder forms—are hallmarks of CKD. In a mouse model, lower PTC density and rounder PTC profiles occurred with experimentally induced IFTA, and computational modeling has suggested rounder PTC profiles may improve homogeneity of tissue oxygenation.^3^ Lower PTC density in cortical interstitium has been shown to be associated with progressive CKD in patients with proteinuric glomerular diseases, but not after adjusting for %IFTA.^1^ In our present study, we found that more circular and less elongated PTC profiles in the non-IFTA cortex were independently associated with progressive CKD after adjusting for chronic histological changes precisely measured with morphometry (including %IFTA). These findings suggest that information is encoded in the microvasculature of nonfibrotic tubulointerstitium that can predict disease progression before structural damage (IFTA) is evident.

There is inherent variability in the quality of needle core biopsies of kidney tissue, with frequent problems from inadequate amount of cortex, glomeruli, or artery sampling.^22^ This sampling error may lead to an inaccurate assessment of the degree of chronicity on kidney histology. Prior work focused on kidney biopsies, but we had access to large wedge sections, which provided a more comprehensive representation of the cortex, including measures of chronic histologic changes (IFTA, arteriosclerosis, and arteriolar hyalinosis) and consistent representation of the medulla across specimens. By studying the microvascular status in both cortex and medulla, we were able to demonstrate that medullary PTC density exhibits similar associations with CKD progression as cortical PTC density. Thus, one could speculate that, in the event of undersampling of cortical tissue, the medullary microvasculature status could be used as a surrogate biomarker of cortical chronicity and disease progression. However, the ability of medullary PTC density to directly estimate cortical %IFTA is limited. Importantly, when cortex is available, medullary PTC density did not contribute to the risk of CKD progression beyond chronic histological changes in the cortex.

There are several reasons why decreased PTC density with rounder and less elongated PTC profiles would be associated with progressive CKD. These morphological changes reflect microvascular reduction (decreased density) and simplification (fewer interconnections that contribute to elongation profiles between tubules) that may impair renal function by reducing oxygen and nutrient delivery to the tubules in regions where IFTA has not yet occurred. This finding aligns with experimental data that show tubular and interstitial fibrogenesis often constricts peritubular capillaries, leading to the loss of perfusion and more circular capillary cross-sections.^3,4^ This corroborates prior observations that fibrotic remodeling constricts peritubular spaces, hindering perfusion and driving more circular cross-sections.^23,24^ A dense and interconnected PTC network is critical in maintaining nutrient and oxygen delivery, reabsorption of fluids and metabolites, and preservation of renal interstitial homeostasis. A reduced PTC density could disrupt these processes, exacerbating renal injury and promoting CKD progression.^25^

Several steps are needed for PTC assessment to be practical in clinical care, which include but are not limited to (1) high-quality digital pathology images for accurately capturing and analyzing histological details; (2) quantification of PTC density in interstitium, requiring the application of models for the segmentation of all other structures (glomeruli, tubules, vessels) such that only interstitium remains; and (3) application of all these models and the extraction of PTC features, requiring substantial expert involvement and high-quality annotated data. Further work is also needed to determine how to implement such models into the workflow of a renal pathology practice. Reference values based on a healthy population (eg, living kidney donors)^26^ are needed (possibly age- and sex-specific) and would aid in the interpretation of the PTC features.

There were several potential limitations to this work to consider. Our study cohort comprised nephrectomy specimens from patients with renal tumors; findings from PTC features in this population may not always be generalizable to other populations with CKD or to clinical practice that is based on kidney biopsies rather than nephrectomies. Because needle biopsies are much smaller than wedge sections and thus less representative of the entire kidney, further analysis is needed to determine the minimum amount of tissue needed to estimate PTC features for reliable prediction of disease progression. Additionally, this study relied on manual annotation of cortex versus medulla and manual annotations for IFTA, which would not be feasible in clinical practice. We were unable to assess PTCs separately between the inner and outer medulla. Finally, the PTC segmentation model was trained on capillaries with open/visible lumen and does not detect PTCs that are collapsed and detectable only by immunohistochemistry markers of endothelium (CD34).^2^ Thus, while CD34 stain may provide a more comprehensive detection of PTCs, the application of computational image analysis on standard histology (PAS-stained) WSIs offers a potentially more practical and less expensive solution for the detection of patent PTCs.

In summary, alterations in PTC morphology, especially in terms of reduced density, shorter major axis, and increased circularity—are associated with CKD and its progression. These findings not only underscore the pathophysiological relevance of PTCs in CKD but also highlight the feasibility of integrating automated DL-based segmentation methods to characterize PTC features from standard kidney histology. Such approaches could ultimately lead to improved risk stratification and personalized management strategies in CKD. Quantification of PTC features has the potential to not only improve prognostic evaluations but also provide therapeutic targets in patients with CKD.^1,2,6,12,24,25^

## Supplementary Material

1 **Figure S1:** Scatterplot of PTC count density versus major axis length in the non-IFTA cortex region.
**Figure S2:** Scatterplot showing negative correlation between PTC count density in the medulla and the IFTA in the cortex.
**Item S1:** Supplementary methods.
**Table S1:** Spearman’s correlations of PTC features in non-IFTA cortex and medulla with each other.

## Figures and Tables

**Figure 1.**
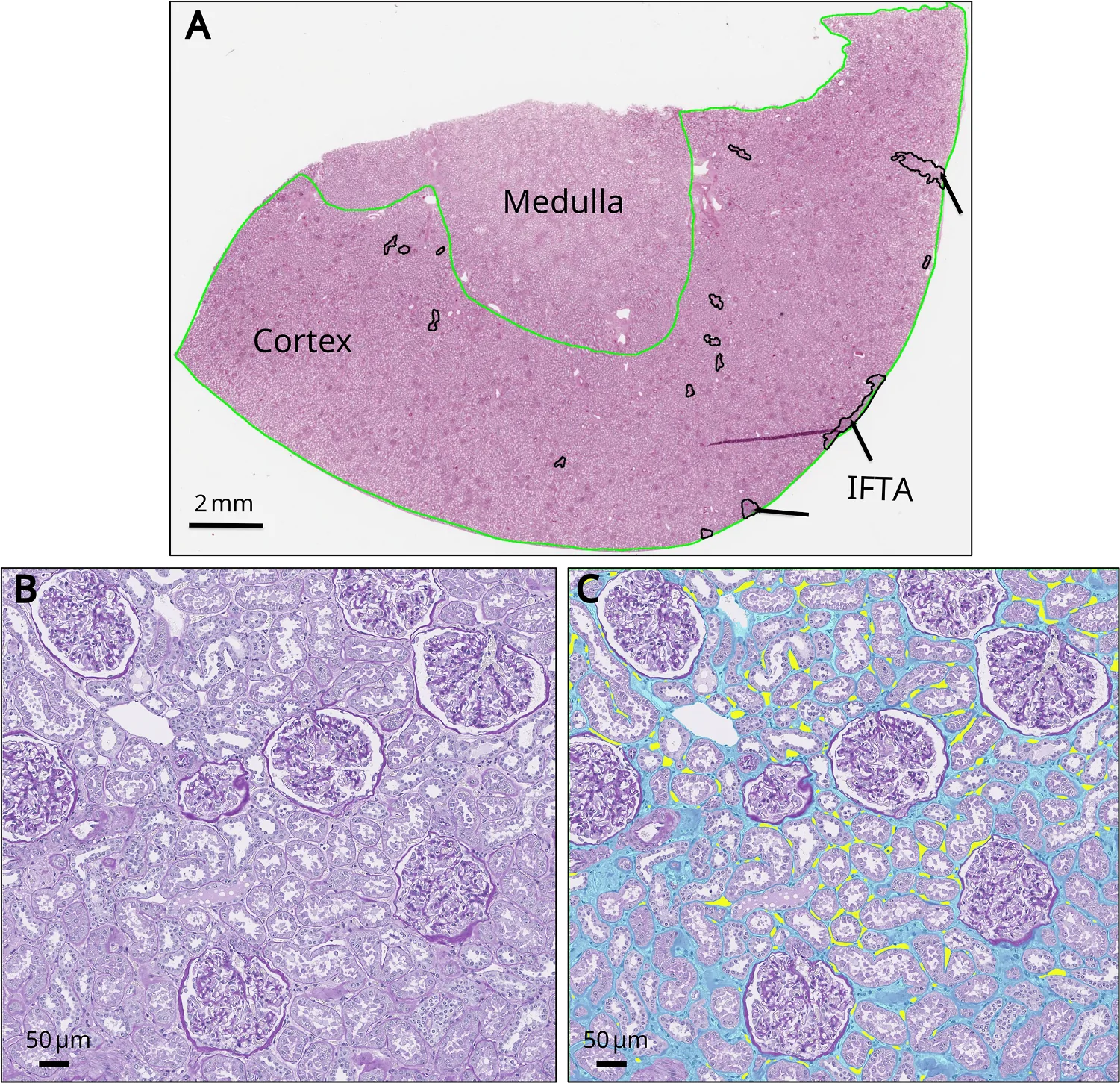
Image analyses obtained on each nephrectomy wedge section. (A) Manually drawn annotation to segment regions of cortex (outlined in green), IFTA (outlined in black) within the cortex, and medulla. (B) Representative PAS-stained non-IFTA cortex. (C) Deep-learning detection of PTCs, highlighted in yellow, within the interstitial fractional space, highlighted in cyan, in the same region. Abbreviation: IFTA, interstitial fibrosis and tubular atrophy.

**Figure 2.**
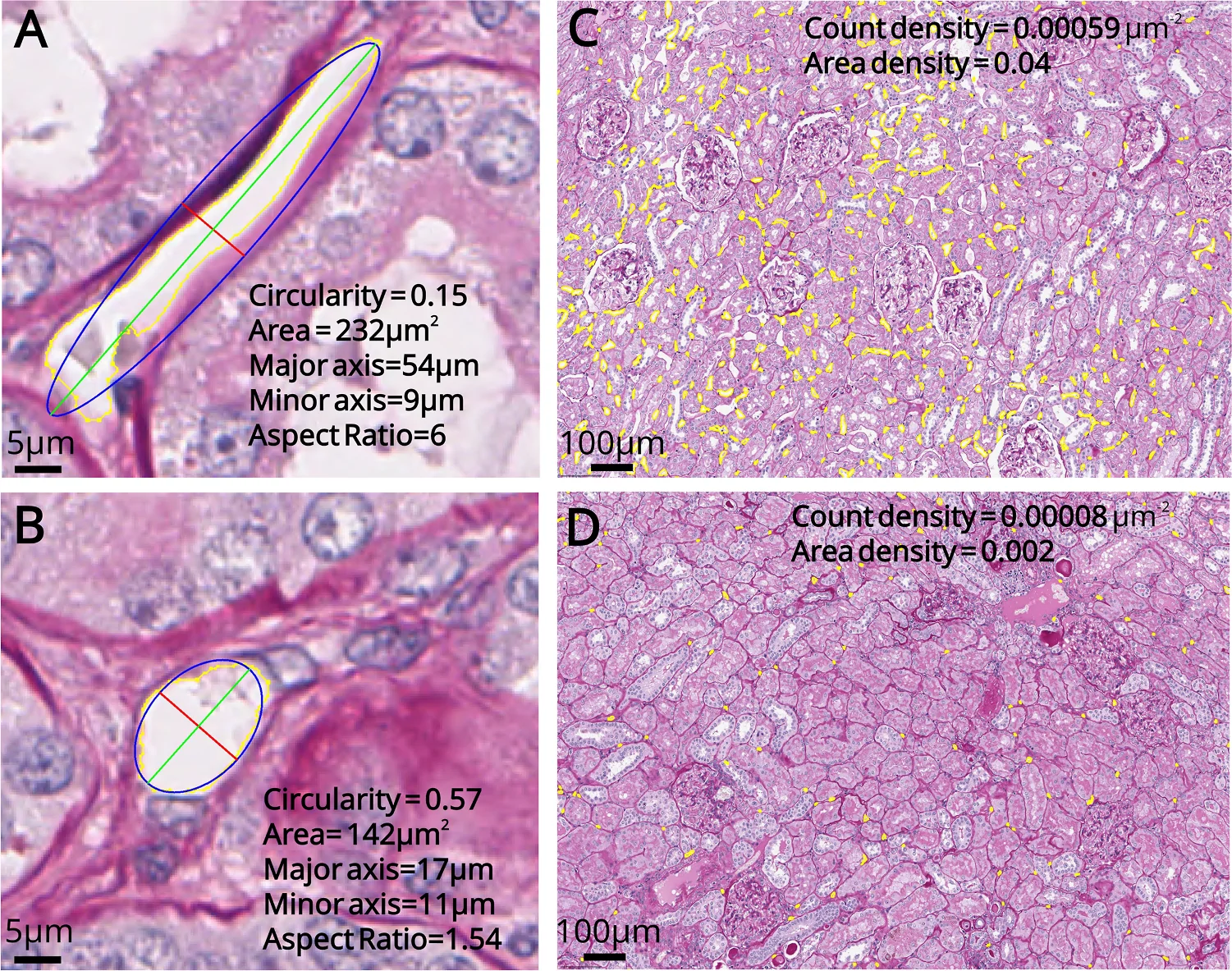
Example of peritubular capillaries (outlined in yellow) and the variability in their features with respect to circularity (blue), area, major axis (green), minor axis (red), and aspect ratio (A-B) and with respect to PTC count and area density (C-D).

**Figure 3.**
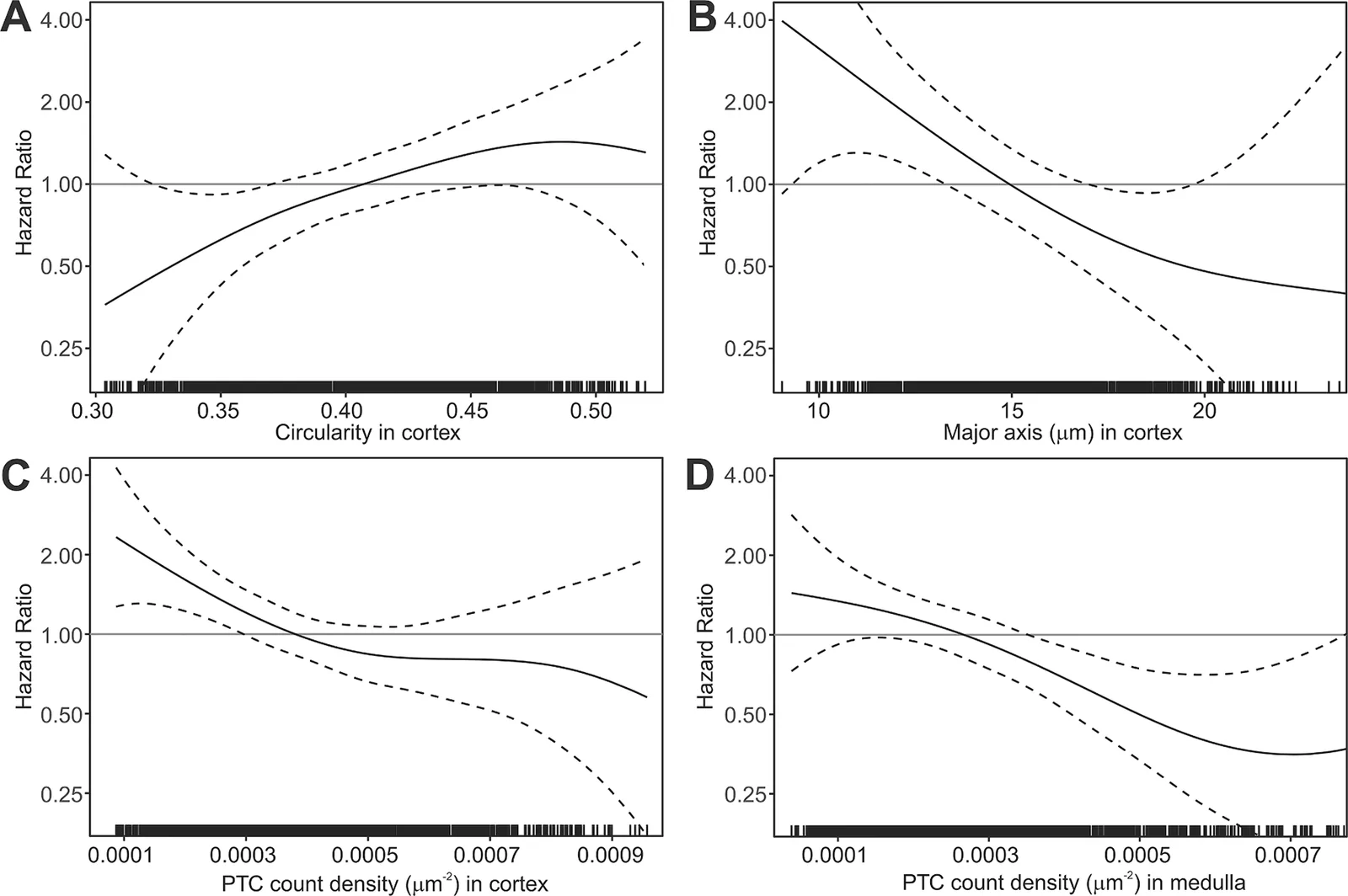
Cubic splines of hazard ratio on a logarithmic scale for progressive chronic kidney disease by PTC (A) circularity, (B) major axis, and (C) count density in non-interstitial fibrosis and tubular atrophy cortex regions, and (D) PTC count density in medulla region. Abbreviation: PTC, peritubular capillary.

**Figure 4.**
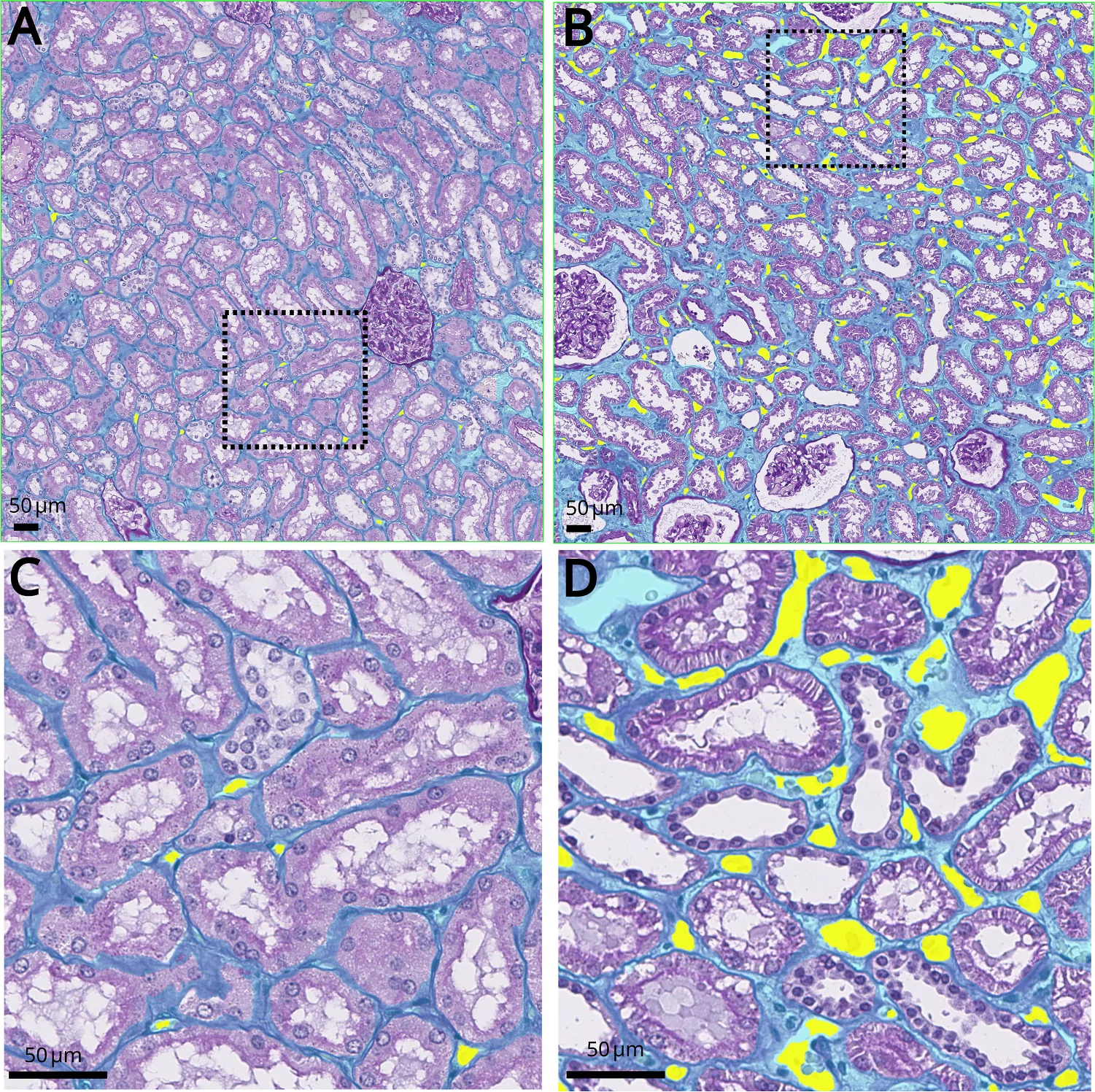
Examples from 2 patients with <5% interstitial fibrosis and tubular atrophy but different PTC patterns. Round, low-density PTCs associated with high risk for progressive CKD are shown in (A) and (C) (PTC area density, 0.007; PTC count density, 0.00014 μm^−2^; and interstitium area, 34,930,290 μm^2^, or 31% of the cortex region). Elongated, high-density PTCs associated with low risk for progressive CKD are shown in (B) and (D) (PTC area density, 0.018; PTC count density, 0.00042 μm^−2^; and interstitium area, 66,565,568 μm^2^, or 36% of the cortex region). (C) and (D) are magnified views of the boxed regions in (A) and (B), respectively. Abbreviations: CKD, chronic kidney disease; PTC, peritubular capillary.

**Table 1.** CKD Risk Factors, Kidney Function, and Chronic Histological Changes at Baseline for the Radical Nephrectomy Patients (n = 1,553)

	Value
Age, y	64 ± 12
Male	1,004 (65%)
BMI, kg/m^2^	31.0 ± 7.1
Diabetes	224 (14%)
Hypertension	1,042 (67%)
Prenephrectomy eGFR, mL/min/1.73 m^2^	73 ± 19
Baseline postnephrectomy eGFR, mL/min/1.73 m^2^	51 ± 14
24-h Urine protein, mg^a^	161 [92–312]
Glomerular volume, mm^3^	0.0029 ± 0.0011
% GSG	8.5% ± 8.9%
% IFTA	3.4% ± 7.2%
IFTA foci density, per cm^2^	24.2 ± 20.6
% Luminal stenosis	53% ± 16%
Arteriolar hyalinosis density, per cm^2^	4.5 ± 8.2

Values for categorical variables are given as percentages; values for continuous variables are given as median [IQR] or mean ± SD.

Abbreviations: BMI, body mass index; CKD, chronic kidney disease; eGFR, estimated glomerular filtration rate; GSG, globally sclerotic glomeruli; IFTA, interstitial fibrosis and tubular atrophy.

a Missing in 254 patients.

**Table 2.** PTC Features for Cortex and Medulla Regions of Histology From Nontumor Sections of Radical Nephrectomy Specimens (n = 1,553)

	Cortex Non-IFTA Regions	Medulla Regions^a^
**Histological Measures**		
PTC count	17,715 ± 11,228	9,937 ± 7,060
PTC total area, μm^2^	999,232 ± 741,505	663,354 ± 560,326
Tissue area, μm^2^	118,192,141 ± 49,047,778	63,209,612 ± 33,489,271
Interstitium area, μm^2^	43,490,075 ± 18,267,603	36,277,472 ± 20,639,684
**PTC Feature**		
Area, μm^2^	54.1 ± 10.9	63.5 ± 15.4
Count density, μm^−2^	0.00041 ± 0.00019	0.00030 ± 0.00017
Area density	0.0249 ± 0.0145	0.021 ± 0.014
Circularity	0.41 ± 0.05	0.46 ± 0.04
Eccentricity	0.87 ± 0.02	0.84 ± 0.02
Major axis, μm	15.4 ± 3.4	14.2 ± 1.8
Aspect ratio	3.0 ± 0.4	2.5 ± 0.4

Values for continuous variables are given as mean ± SD.

Abbreviations: IFTA, interstitial fibrosis and tubular atrophy; PTC, peritubular capillary.

a Analysis limited to 1,491 patients with available medulla.

**Table 3.** Risk of Progressive CKD With PTCs in the Cortex and Medulla of Kidney Histology Wedge Sections

Peritubular Capillary Features per SD	Unadjusted		Adjusted for Chronic Histologic Changes in Cortex		Adjusted for CKD Risk Factors and Kidney Function	
	HR (95% CI)	*P* Value	HR (95% CI)	*P* Value	HR (95% CI)	*P* Value

**Cortex Non-IFTA Regions (n = 1,553)**						
Area	0.78 (0.64–0.97)	0.02	0.98 (0.79–1.22)	0.9	0.88 (0.71–1.09)	0.2
Count density	0.73 (0.59–0.91)	0.005	0.89 (0.72–1.11)	0.3	0.78 (0.62–0.97)	0.03
Area density	0.74 (0.59–0.93)	0.009	0.93 (0.74–1.16)	0.5	0.81 (0.65–1.02)	0.07
Circularity	1.30 (1.07–1.60)	0.01	1.26 (1.02–1.57)	0.03	1.30 (1.05–1.61)	0.02
Eccentricity	0.90 (0.73–1.10)	0.3	0.88 (0.71–1.09)	0.3	0.89 (0.72–1.10)	0.3
Major axis	0.52 (0.37–0.75)	<0.001	0.68 (0.49–0.96)	0.03	0.60 (0.42–0.87)	0.007
Aspect ratio	0.88 (0.71–1.09)	0.2	0.84 (0.69–1.02)	0.08	0.87 (0.70–1.08)	0.2
**Medulla Regions (n = 1,491)**						
Area	0.88 (0.71–1.09)	0.2	0.85 (0.68–1.06)	0.1	0.85 (0.68–1.06)	0.2
Count density	0.71 (0.56–0.91)	0.006	0.89 (0.69–1.15)	0.4	0.79 (0.61–1.02)	0.07
Area density	0.68 (0.53–0.88)	0.004	0.82 (0.62–1.08)	0.2	0.73 (0.56–0.96)	0.02
Circularity	0.97 (0.79–1.21)	0.8	1.00 (0.80–1.25)	0.9	0.99 (0.79–1.23)	0.9
Eccentricity	0.98 (0.78–1.22)	0.8	0.92 (0.73–1.15)	0.4	0.97 (0.78–1.21)	0.8
Major axis	0.84 (0.67–1.06)	0.1	0.77 (0.61–0.97)	0.03	0.82 (0.64–1.04)	0.09
Aspect ratio	1.04 (0.83–1.29)	0.8	0.95 (0.75–1.20)	0.7	1.00 (0.80–1.25)	0.9

Chronic histologic changes in cortex were percentage globally sclerotic glomeruli, percentage of IFTA, IFTA foci density, percentage luminal stenosis, and arteriolar hyalinosis density. CKD risk factors and kidney function were age, sex, body mass index, hypertension, diabetes mellitus, estimated glomerular filtration rate, and proteinuria.

Abbreviations: CKD, chronic kidney disease; HR, hazard ratio; IFTA, interstitial fibrosis and tubular atrophy; PTC, peritubular capillary.

**Table 4.** Spearman’s Correlations of Peritubular Capillary Features in Non-IFTA Cortex With Clinical and Histologic Characteristics Among the 1,553 Patients

	Count Density		Area Density		Major Axis		Circularity	
	*r*_s_	*P* Value	*r*_s_	*P* Value	*r*_s_	*P* Value	*r*_s_	*P* Value
**CKD Risk Factors and Kidney Function**								
Age	−0.06	0.01	−0.09	<0.001	−0.08	0.002	0.06	0.01
Male	−0.09	<0.001	−0.03	0.3	0.10	<0.001	−0.10	<0.001
BMI	−0.08	0.003	−0.09	<0.001	−0.07	0.01	−0.05	0.03
Diabetes mellitus	−0.03	0.3	−0.03	0.3	−0.01	0.7	−0.01	0.8
Hypertension	−0.11	<0.001	−0.13	<0.001	−0.09	<0.001	−0.02	0.5
Prenephrectomy eGFR	0.08	0.001	0.11	<0.001	0.16	<0.001	−0.16	<0.001
Proteinuria	−0.14	<0.001	−0.13	<0.001	−0.11	<0.001	0.09	0.002
**Chronic Histological Changes**								
Glomerular volume	−0.15	<0.001	−0.12	<0.001	0.02	0.3	−0.14	<0.001
% GSG	−0.13	<0.001	−0.18	<0.001	−0.14	<0.001	0.04	0.2
% IFTA	−0.20	<0.001	−0.23	<0.001	−0.19	<0.001	0.07	0.009
IFTA foci density	−0.11	<0.001	−0.14	<0.001	−0.14	<0.001	0.05	0.05
% Luminal stenosis	−0.08	0.001	−0.13	<0.001	−0.11	<0.001	0.02	0.4
Arteriolar hyalinosis density	−0.10	<0.001	−0.07	0.008	0.01	0.8	0.01	0.7

Abbreviations: BMI, body mass index; CKD, chronic kidney disease; eGFR, estimated glomerular filtration rate; GSG, globally sclerotic glomeruli; IFTA, interstitial fibrosis and tubular atrophy.

## Data Availability

Clinical data can only be shared with a data use agreement and Institutional Review Board approval.

## PLAIN-LANGUAGE SUMMARY

Peritubular capillaries support oxygen transport, nutrient exchange, and waste elimination by the renal tubules in the kidney. Using artificial intelligence and image processing tools, we characterized the size, shape, and density of these capillaries in sections of human kidneys removed from patients with a kidney tumor. The study found that in the nonscarred regions of kidney tissue, lower density and rounder capillaries in the space between tubules were associated with a subsequent loss of kidney function.
