# Supplementary material for: Association of Histological Features of Cortical and Medullary Peritubular Capillaries With Progressive CKD
Source: Am J Kidney Dis. Author manuscript; Available in PMC 2026 Oct 14. (PMC13660368; doi:10.1053/j.ajkd.2026.05.010)
Supplement: Supplementary file 1 [file NIHMS2212555-supplement-1.pdf]

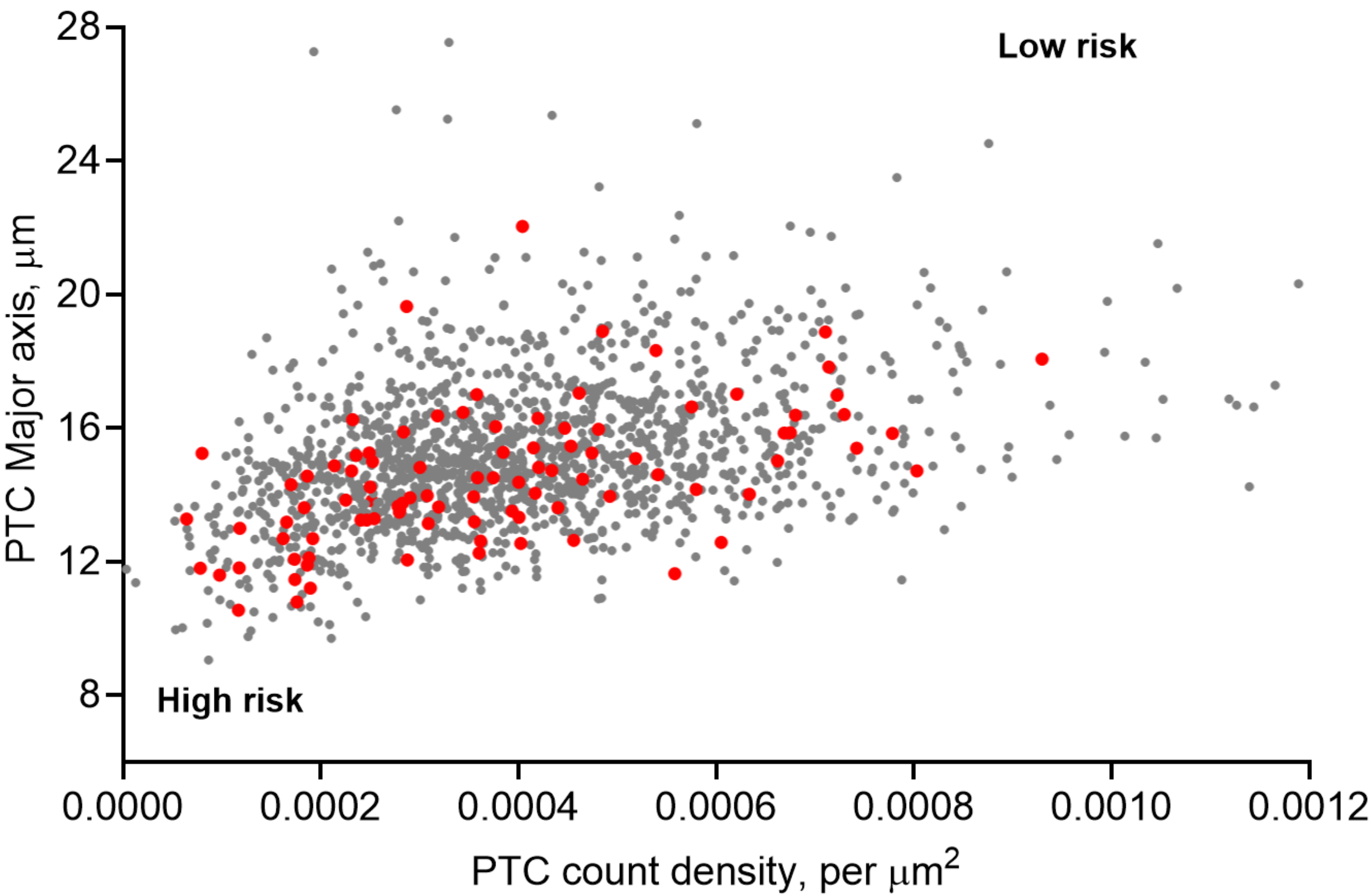

Figure S1. Scatterplot of PTC count density versus major axis length in the non-IFTA cortex region. Persons with shorter PTC major axis and lower PTC count density were at high risk for progressive CKD (red dots) than persons with longer PTC major axis and higher PTC count density.

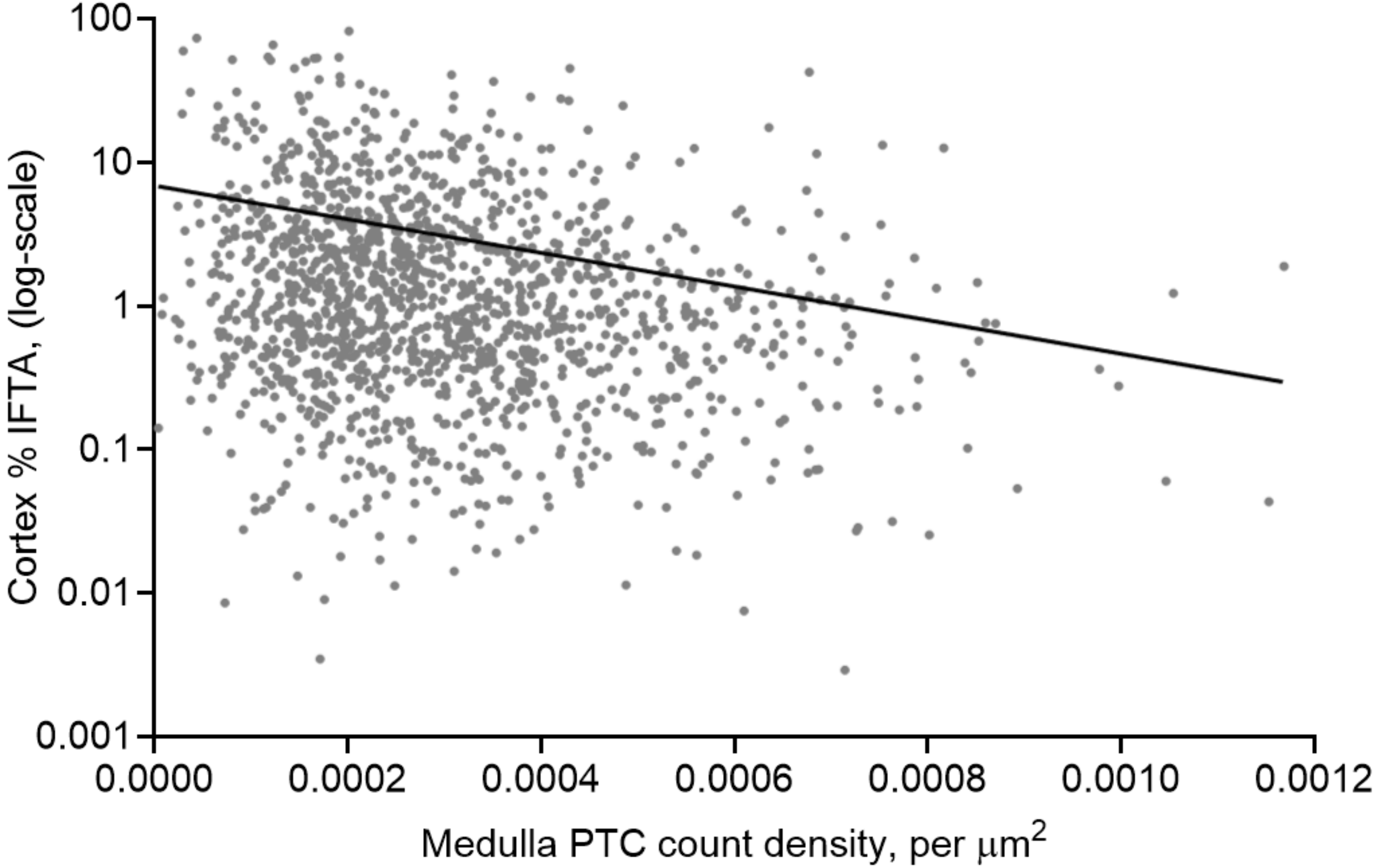

Figure S2. Scatter plot showing negative correlation between peritubular capillary (PTC) count density in the medulla (x-axis) and the interstitial fibrosis and tubular atrophy (IFTA) in the cortex (y-axis). The solid line denotes the regression line (r_s= -0.19, p<0.0001).

Item S1. Supplementary Methods

Formulas for Quantifying PTC attributes

1. PTC Eccentricity:

Eccentricity quantifies the best fit ellipse for a PTC’s deviation from a circle.

$$Eccentricity = \sqrt{(1 - \left(\frac{Minor\ Axis\ Length}{Major\ Axis\ Length}\right)^2}$$

Values range from 0 (a perfect circle) to values approaching 1 (a highly elongated shape).

2. Aspect Ratio (AR):

The aspect ratio is defined as the ratio of the major axis to the minor axis of the best-fit ellipse for a PTC.

$$Aspect\ ratio = \frac{Major\ Axis\ Length}{Minor\ Axis\ Length}$$

3. PTC Circularity:

Circularity measures how close the shape is to a perfect circle.

$$Circularity = \left(\frac{4\pi\ X\ Area}{Perimeter^2}\right)$$

A value of 1 indicates a perfect circle, with lower values indicating deviations from circularity.

4. PTC Count Density:

This attribute reflects the number of PTCs per unit area of the region.

$$Count\ Density = \frac{Number\ pf\ PTCs}{Area\ of\ Region}$$

As this was measured in the interstitium, it is calculated as:

$$Count\ Density\ in\ the\ interstitium = \frac{Number\ of\ PTCs\ in\ interstitium}{Area\ of\ insterstitium}$$

5. PTC Area Density:

This attribute represents the total area occupied by PTCs per unit area of the region.

$$Area\ Density = \frac{Total\ PTC\ Area}{Area\ of\ Region}$$

As this was measured in the interstitium, it is calculated as:

$$Area\ Density\ in\ the\ interstitium = \frac{Total\ PTC\ Area\ in\ interstitium}{Area\ of\ interstitium}$$

Table S1. Spearman's correlations of peritubular capillary (PTC) attributes in non-IFTA cortex and medulla with each other.

	**PTC count density**		**PTC area density**		**PTC major Axis**		**PTC circularity**	
	**r_s**	**P value**	**r_s**	**P value**	**r_s**	**P value**	**r_s**	**P value**
**Peritubular capillary attributes in non-IFTA cortex regions**								
Count density	---	---	0.96	<0.001	0.37	<0.001	0.04	0.2
Area density	0.96	<0.001	---	---	0.48	<0.001	0.07	0.004
Circularity	0.04	0.2	0.07	0.004	-0.47	<0.001	---	---
Eccentricity	-0.02	0.4	-0.04	0.1	0.59	<0.001	-0.70	<0.001
Major axis	0.37	<0.001	0.48	<0.001	---	---	-0.47	<0.001
Aspect ratio	-0.05	0.07	-0.07	0.007	0.61	<0.001	-0.70	<0.001
**Peritubular capillary attributes in medulla regions**								
Count density	---	---	0.94	<0.001	0.16	<0.001	0.25	<0.001
Area density	0.94	<0.001	---	---	0.38	<0.001	0.25	<0.001
Major axis	0.16	<0.001	0.38	<0.001	---	---	-0.40	<0.001
Circularity	0.25	<0.001	0.25	<0.001	-0.40	<0.001	---	---
Eccentricity	-0.21	<0.001	-0.27	<0.001	0.35	<0.001	-0.80	<0.001
Aspect ratio	-0.27	<0.001	-0.33	<0.001	0.34	<0.001	-0.77	<0.001
